# Early Haematologic Response to Oral Iron Treatment in Adults With Moderate Iron‐Deficiency Anaemia

**DOI:** 10.1155/anem/8866390

**Published:** 2025-11-20

**Authors:** Patrice Cacoub, Fredrick O. Otieno, Julie Trichereau, Océane Gobert, Anne-Laure Kerveillant, Julie Escola, François Verrière

**Affiliations:** ^1^ Department of Internal Medicine and Clinical Immunology, La Pitié-Salpêtrière Hospital, Sorbonne Université, Paris, France, sorbonne-universites.fr; ^2^ Nyanza Reproductive Health Society, Kisumu, Kenya; ^3^ ICTA, Fontaine-lès-Dijon, France; ^4^ Innotech International, Arcueil, France

**Keywords:** anaemia, copper, ferritin levels, ferrous gluconate, haemoglobin, iron deficiency anaemia, manganese, oral iron supplementation, serum iron, Tothema

## Abstract

**Background:**

Identifying early response to oral iron supplementation in anaemic patients is key. This study aims to assess the onset‐of‐action of oral liquid solution of ferrous gluconate, copper and manganese gluconate in adults with moderate iron deficiency anaemia.

**Methods:**

This prospective, open‐label study was conducted in France, Bulgaria and Kenya. All patients received 150 mg of oral ferrous gluconate/day for 12 weeks. Eleven blood samples were taken at Day 7 and at Days 0, 3, 5, 7, 10, 14, 21, 28, 56 and 84. The primary endpoint was to assess treatment duration required to observe a +0.5 g/dL haemoglobin (Hb) level increase.

**Results:**

Ninety‐five moderately anaemic patients were included in the analysis. Hb increase ≥ 0.5 g/dL was observed within 9‐10 days of treatment (0.51 g/dL at Day 10, 95% CI: 0.45–0.57 and *p* < 0.0001). Serum iron increased within the first 3 days (mean change from baseline: 67.21 ± 91.57 μg/dL). An improvement of quality of life (physical component mean change: 8.20 ± 7.34 and mental component: 6.72 ± 6.67) was reported after treatment. The safety profile was very good.

**Conclusion:**

This study demonstrates for the first time the rapid onset‐of‐action of oral liquid solution of ferrous gluconate and confirms its good tolerability.

**Trial Registration:**

ClinicalTrials.gov identifier: NCT04309669

## 1. Introduction

Anaemia, defined as haemoglobin (Hb) levels lower than 12.0 g/dL in females and 13.0 g/dL in males [1, 2], is a major public health issue with 1.92 billion people affected globally [3]. It is caused by iron deficiency in over 50% of the cases. Iron stores are assessed by measuring ferritin levels, which typically range from 20 to 200 μg/L in women and from 30 to 300 μg/L in men [4]. Iron deficiency arises from factors such as inadequate dietary iron intake/absorption, increased iron needs during pregnancy or growth periods, increased iron loss, heavy menstrual bleeding, chronic inflammatory diseases or helminth infestation [4].

Apart from investigating and managing the cause of iron loss, there are three options for the treatment of iron deficiency anaemia (IDA): oral iron therapy, intravenous iron administration or blood transfusion [5]. Transfusion, even when allogeneic, autologous or restrictive is not free from constraints, contraindications and risks [6]. Consequently, apart from emergency cases where transfusion is necessary, oral iron supplementation and intravenous iron preparations are the preferred approaches to treat IDA with the aim to replenish iron stores [7]. The choice between these two latter options does not necessarily depend on Hb levels but on the clinical impact of the anaemia, the patient’s ability to respond rapidly to treatment and the risks associated with respective routes of administration.

Oral iron supplementation is the first line treatment in most cases due to its ease of administration, accessibility and efficacy, but it can cause gastrointestinal side effects such as constipation, diarrhoea and abdominal pain. A meta‐analysis of 21 studies on 4745 patients concluded that, compared with placebo, oral supplementation in IDA significantly improves Hb levels and probably reduces the need for blood transfusion [8].

Intravenous iron supplementation can be preferred when rapid correction of iron deficit is required, but it is far more expensive and exposes the patient to safety issues, such as risk of infection [9], infusion reaction [10–12], hypophosphatemia [13] and acute hypersensitivity reactions which, although very rare, can be life‐threatening [14]. The French National Agency for Medicines and Health Products Safety (ANSM) has recently published additional risk minimization measures concerning the risks of hypersensitivity and anaphylactic shock associated with these treatments [15].

In current clinical practice, response to oral iron therapy is often assessed after 1 month of treatment. The management of patients suffering from IDA could be improved if response to treatment was detected earlier. Indeed, from a clinical perspective, identifying an early response to treatment is the key to confirm the clinical benefit of oral iron supplementation. Conversely, detecting treatment failure early could allow a quick readjustment of treatment.

In a review of five trials on oral iron supplementation in IDA, 72.8% of the patients were classified as responders after 14 days of treatment (Hb increase ≥ 1 g/dL) [16]. Such results were mainly reported with iron sulphate; no clinical data are available for ferrous gluconate. Although a randomized trial in children showed comparable prophylactic efficacy between ferrous sulphate and ferrous gluconate [17], data on treatment response kinetics in IDA in adults remain lacking. Available studies most often assess either the bioavailability profile of serum iron after a single iron intake [18, 19] or the efficacy of ferrous gluconate supplementation over several weeks by measuring Hb and ferritin levels [20, 21] without addressing treatment response time. To assess the kinetics of efficacy biomarkers of iron supplementation, data from repeated biological iron assays are required, especially during the first week of treatment.

In this context, the aim of the FAST study (Assessments of the eFficacy, the onset‐of‐Action and the Safety of Tot’héma in adults with moderate iron deficiency anaemia) was to assess the kinetics of the main iron biological parameters from the very early stages of treatment with an oral liquid solution of ferrous gluconate combined with copper and manganese, in adults with moderate IDA, and to evaluate the onset of action of this treatment.

## 2. Materials and Methods

The FAST study was a multicentre, open‐label, prospective, noncomparative study conducted from November 2020 to October 2023 at twelve participating sites located in Europe (France and Bulgaria) and Africa (Kenya). Participants were treated over a 12‐week period and assessed during five visits: a screening visit performed within seven days prior to the inclusion (V1/D‐7 to D‐1), an inclusion visit (V2/D0), two follow‐up visits on Day 14 (V3/D14) and Day 28 (V4/D28) and one end of study visit on Day 84 (V5/D84). Eleven blood investigations were performed from the screening period until the end of the study.

Participants were treated with a ferrous gluconate (Fe) hydrate, copper gluconate (Cu) and manganese gluconate (Mn) oral liquid solution (Tothema [50 mg Fe, 0.70 mg Cu, 1.33 mg Mn], Laboratoire INNOTECH INTERNATIONAL) for 12 weeks (84 days). Participants received one ampoule three times a day, for a total of 150‐mg iron as ferrous gluconate per day.

Eligible participants were patients with confirmed moderate anaemia (Hb levels between 8 g/dL and 10 g/dL [limits included]) and depleted ferritin stores (ferritin blood level below the lower limit of normal laboratory values) at screening. Main medical exclusion criteria were any evidence of underlying diseases such as acute digestive bleeding, or acute/chronic inflammatory syndrome; acute malaria crisis within 15 days prior to inclusion; gastrointestinal disorders incompatible with study treatment compliance and C‐reactive protein above 10 mg/L at screening.

At each visit, the investigator performed physical examinations and assessed levels of fatigue with a Visual Analogue Scale (VAS) (ranging from 0 [*not at all tired*] to 10 [*extremely tired*]). Quality of life was evaluated at inclusion and at the end of study with the 36‐item short form survey instrument (SF‐36) questionnaire for participants included in Europe only [22]. The SF‐36 measures eight health domains with 36 questions, transformed to scores ranging from 0, representing poor health status, to 100, representing optimal health status. The domains are then combined into two summary scores: the physical and the mental component summary scores. Blood investigations were performed from the screening period until the end of study visit. Hb levels, serum iron, reticulocytes, transferrin saturation (T‐SAT) and mean corpuscular volume (MCV) were measured at screening, D0, D3, D5, D7, D10, D14, D21, D28, D56 and D84. Ferritin blood levels were measured at the same time points except at D3, D5 and D10. Laboratory analyses were performed at a certified local laboratory. In addition, to ensure the reliability of the results, all sample analyses for a given participant were performed in the same laboratory.

The primary objective was to determine the duration of treatment with oral ferrous gluconate needed to observe an Hb increase of 0.5 g/dL from the baseline value. The primary endpoint was the first time (in days) associated with a mean Hb increase of at least 0.5 g/dL. Secondary endpoints included at each sampling time point: proportion of participants presenting an Hb increase ≥ 0.5 g/dL, ≥ 1 g/dL and ≥ 2 g/dL; proportion of participants with normal Hb levels (Hb ≥ 12 g/dL for women and Hb ≥ 13 g/dL for men); change in Hb, serum iron, ferritin, reticulocytes, T‐SAT and MCV over time and, at each applicable time point, change in fatigue and quality of life.

All statistical analyses were performed using Statistical Analysis Systems (SAS) Version 9.4.

The safety analysis included all enrolled participants who took at least one dose of the study treatment. The full analysis set corresponds to patients in the safety analysis, in which only those with postinclusion data are retained.

Continuous variables were summarised descriptively with the mean, 95% confidence interval (CI), standard deviation (SD), median, first and third quartile (Q1, Q3), range, minimum and maximum. Frequencies and percentages were reported for categorical variables. No systematic statistical testing was performed. Subgroup analyses were performed according to geographic zone (Europe/Africa). For the primary endpoint, a linear mixed model for repeated measures with random effects was fitted to observed data without any imputation of missing data to analyse the change in Hb over the 12‐week follow‐up period. The model included the following variables: change in Hb level from D0 (dependent variable), geographic zone (fixed effect), real time point Di (random effect) and interaction term for geographic zone by time interaction. The unstructured covariance matrix was used to model random effects. Time points were treated as a continuous variable using real time points. Hb changes from D0 predicted by the model at each real time point (from D1 to D10 by 1 and from D10 to D90 by 10) were estimated using the REstricted Maximum Likelihood (REML) method. The 95% CIs were produced, and the first time associated with a predicted mean Hb increase of at least 0.5 g/dL was identified. Time linearity and model validity were checked via graphic representations and residual analysis, respectively. Time to an Hb increase of 0.5 g/dL and 1 g/dL level (time to onset [in days]) was analysed using the Kaplan–Meier method, providing a two‐sided 95% CI for median survival time.

Power calculations were based on the detection of a significant increase in mean Hb level of 0.5 g/dL, using a SD of 1.5 g/dL, with a power of 90% and a two‐sided alpha risk of 0.05, required a total of 97 evaluable participants. Considering a 20% rate of nonevaluable participants, 121 participants were planned to be enrolled. All tests were two‐tailed tests, and *p* < 0.05 was considered significant.

The protocol was approved by the competent authorities of France (Agence Nationale de Sécurité du Médicament et des produits de santé), Bulgaria (Bulgarian Drug Agency) and Kenya (Pharmacy and Poisons Board) and by independent ethics committee from the three countries (Comité de Protection des Personnes, Poitiers Ouest III, France; Ethics Committee for Clinical Trials, Bulgaria; Jaramogi Oginga Odinga Teaching and Referral Hospital, Institutional Scientific and Ethical Research Committee, Kenya). The study was conducted in accordance with the protocol, the Declaration of Helsinki and its amendments, the applicable International Conference of Harmonization E6(R2) Good Clinical Practices Guidelines and the applicable local regulations. All participants provided written informed consent before study inclusion.

Permission was obtained from the copyright holder to use the SF‐36 questionnaire.

Anonymized data not published within this article will be made available by request from any qualified investigator.

## 3. Results

**Figure 1:**
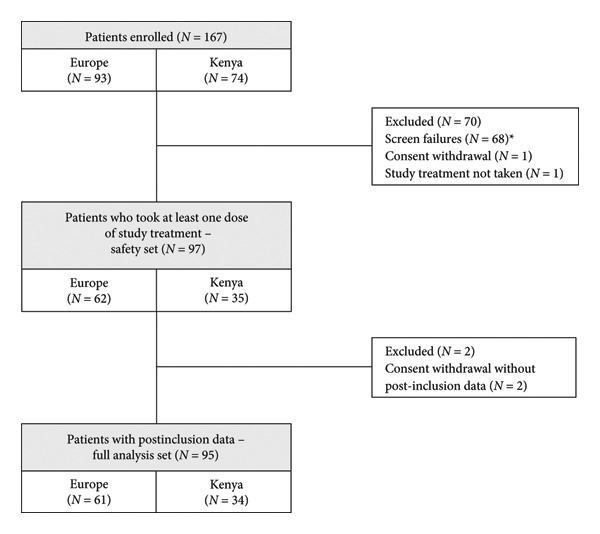
Study flowchart. ^∗^68 screen failures as their laboratory results at screening did not confirm the diagnosis of moderate anaemia with depleted ferritin stores. *N*: number of participants.

Treatment was initiated by 97 participants and data were available for analysis for 95 patients (Figure 1). The study period was completed by 91 participants as four patients discontinued early. Characteristics of the study participants are detailed in Table 1. Mean age was 41.91 years (range: 18.7–83.9) and 87.4% of the participants were women. At the inclusion visit, mean Hb was 9.13 g/dL, mean serum iron was 29.88 μg/dL, mean ferritin blood level was 17.85 ng/mL and mean reticulocyte count was 1.38%. Most participants had symptoms associated with IDA such as conjunctival pallor (73.4% of the participants), fatigue (mean VAS: 4.71) and impaired quality of life (mean SF‐36 summary score, physical component: 46.19 and mental component: 46.39).

**Table 1:** Characteristics of study participants.

	Geographic zone		
	Europe *N* = 61	Kenya *N* = 34	Total *N* = 95
Demographics			
Age, mean ± SD	51.26 ± 18.13	25.14 ± 5.35	41.91 ± 19.45
Female, *n* (%)	51 (83.6%)	32 (94.1%)	83 (87.4%)
Laboratory results at baseline (V2/D0)			
Haemoglobin (g/dL), mean ± SD	9.43 ± 0.97	8.58 ± 0.64	9.13 ± 0.96
Serum iron (μg/dL), mean ± SD	35.75 ± 20.81	19.35 ± 9.38	29.88 ± 19.23
Ferritin blood level (ng/mL), mean ± SD	24.51 ± 60.22	5.91 ± 3.96	17.85 ± 49.00
Ferritin blood level (ng/mL), median (Q1‐Q3)	10.00 (6.50–17.10)	5.00 (4.00–7.00)	7.00 (5.00–12.90)
Reticulocytes (%), mean ± SD	1.65 ± 1.27	0.95 ± 0.32	1.38 ± 1.07
MCV (fL), mean ± SD	73.66 ± 13.73	65.41 ± 5.64	70.65 ± 12.09
T‐SAT (%), mean ± SD	10.75 ± 11.13	3.70 ± 1.86	8.17 ± 9.54
CRP (mg/L), mean ± SD	3.98 ± 5.95	1.71 ± 2.52	3.15 ± 5.08
Conjunctival pallor at baseline (V2/D0), n/N^∗^ (%)			
Present	35/60 (58.3%)	34/34 (100.0%)	69/94 (73.4%)
Fatigue assessment VAS at baseline (V2/D0), *N*	58	34	92
VAS, mean ± SD	5.24 ± 2.34	3.79 ± 0.95	4.71 ± 2.06
Quality of life at baseline (V2/D0), *N*	48		48
Physical component summary score, mean ± SD	46.19 ± 7.72		46.19 ± 7.72
Mental component summary score, mean ± SD	46.39 ± 7.25		46.39 ± 7.25

Abbreviations: CRP, C‐reactive protein; D, day; MCV, mean corpuscular volume; T‐SAT, transferrin saturation; V, visit; VAS, Visual Analogue Scale.

^∗^n/N: number of participants/total number of participants with data available for analysis (modalities with missing data).

From the laboratory results recorded at the scheduled time points and the modelling of the change in mean Hb level between D0 and D90, it was estimated that a significant increase in Hb from baseline (*p* < 0.0001) and an Hb increase of at least 0.5 g/dL can be observed within 9‐10 days of treatment (estimate at D10: 0.51, 95% CI: 0.45–0.57 and *p* < 0.0001) for the overall study population (Table 2). Similar results were found in both geographic zones.

**Table 2:** Primary endpoint: changes in haemoglobin (g/dL) from baseline.

	Hb estimate	Standard error	Lower confidence interval	Upper confidence interval	*p* value
Overall (*N* = 95)					
Time = D9	0.4633	0.02784	0.4080	0.5185	< 0.0001
Time = D10	0.5147	0.03093	0.4533	0.5762	< 0.0001
Time = D39	2.0074	0.1206	1.7678	2.2470	< 0.0001
Kenya (*N* = 34)					
Time = D8	0.4602	0.03802	0.3828	0.5375	< 0.0001
Time = D9	0.5177	0.04278	0.4306	0.6047	< 0.0001
Time = D35	2.0132	0.1664	1.6747	2.3516	< 0.0001
Europe (*N* = 61)					
Time = D11	0.4997	0.04143	0.4168	0.5825	< 0.0001
Time = D12	0.5451	0.04520	0.4547	0.6355	< 0.0001
Time = D45	2.0441	0.1695	1.7051	2.3832	< 0.0001

*Note:* D, day; Hb, haemoglobin; N, number of participants with data available for analysis.

Evolution of Hb, serum iron, ferritin and reticulocytes at each sampling time point are illustrated in Figure 2, and changes in all laboratory markers are detailed in Table 3. After treatment initiation, serum iron levels quickly rose, reaching a peak at D7. Mean Hb levels increased from 9.13 to 12.46 g/dL at the end of the study. Reticulocyte production increased in parallel with a peak observed at D10 (mean change from D0, 1.09%). Mean ferritin blood levels increased from 17.85 to 42.76 ng/mL at the end of the study.

Figure 2Laboratory parameters evolution by visit (serum iron, reticulocyte, haemoglobin, blood ferritin). (a) Serum iron evolution by visit; (b) Reticulocytes evolution by visit; (c) haemoglobin evolution by visit and (d) blood ferritin evolution by visit. Within each box, horizontal lines denote median values and diamonds (or big circle or triangle, for the bottom plot) represent the mean; boxes extend from the 25th to the 75th percentile of each group’s distribution of values; vertical extending lines denote adjacent values (i.e., the most extreme values within 1.5 interquartile range of the 25th and 75th percentile of each group); circles denote outliers outside the range of adjacent value.

**Figure:**
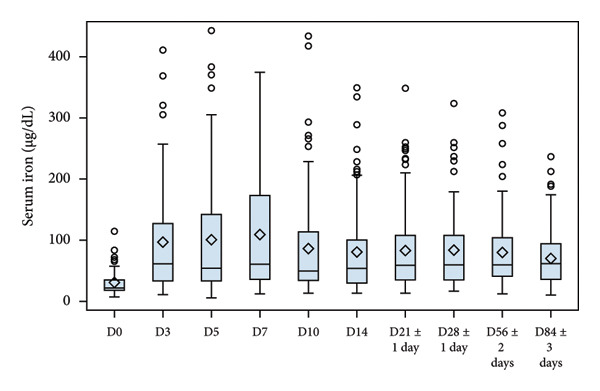
(a)

**Figure:**
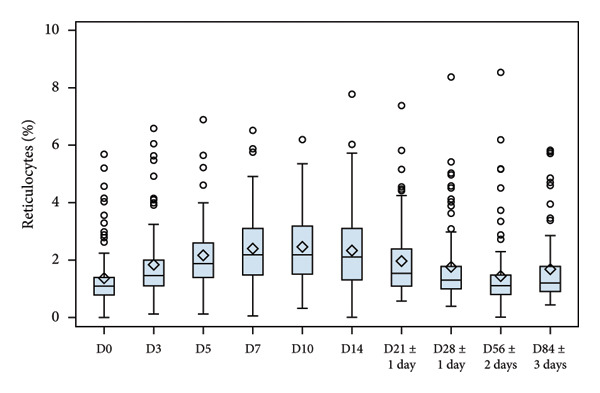
(b)

**Figure:**
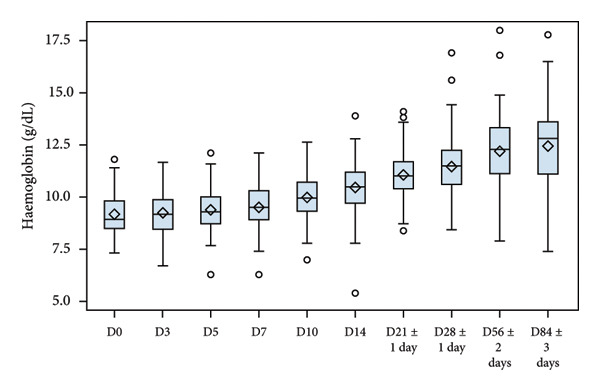
(c)

**Figure:**
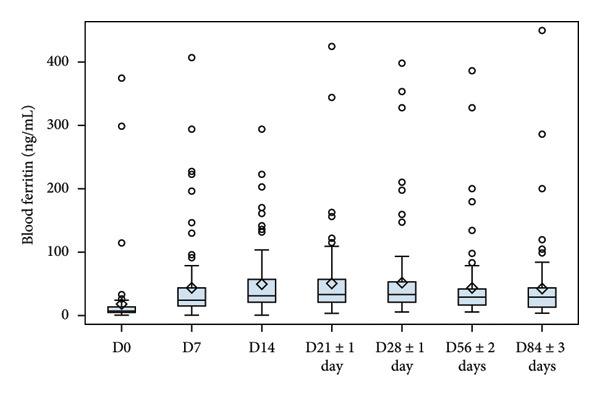
(d)

**Table 3:** Laboratory parameters evolution by visit.

	V2/D0	D3	D5	D7	D10	V3/D14	D21	V4/D28	D56	V5//D84
Serum iron (μg/dL)										
*N*	95	92	90	94	93	93	89	88	86	88
Mean ± SD	29.88 ± 19.23	96.55 ± 88.00	100.58 ± 97.02	109.44 ± 96.46	86.59 ± 83.83	81.06 ± 74.39	84.35 ± 69.38	85.36 ± 68.69	79.33 ± 60.98	70.96 ± 46.51
Reticulocytes (%)										
*N*	88	87	88	89	90	91	88	84	85	86
Mean ± SD	1.38 ± 1.07	1.85 ± 1.25	2.16 ± 1.15	2.40 ± 1.25	2.46 ± 1.23	2.32 ± 1.34	1.96 ± 1.23	1.78 ± 1.33	1.41 ± 1.26	1.68 ± 1.47
Haemoglobin (g/dL)										
*N*	95	92	92	94	94	95	90	88	86	88
Mean ± SD	9.13 ± 0.96	9.24 ± 0.98	9.41 ± 0.95	9.53 ± 0.97	10.00 ± 0.96	10.46 ± 1.23	11.06 ± 1.17	11.50 ± 1.40	12.24 ± 1.75	12.46 ± 1.85
Increase in Hb level ≥ 0.5 g/dL *n* (%)^∗^		18 (19.6%)	37 (40.2%)	47 (50.0%)	68 (72.3%)	77 (81.1%)	79 (87.8%)	81 (92.0%)	77 (89.5%)	80 (90.9%)
Increase in Hb level ≥ 1 g/dL *n* (%)^∗^		7 (7.6%)	11 (12.0%)	17 (18.1%)	41 (43.6%)	64 (67.4%)	68 (75.6%)	73 (83.0%)	72 (83.7%)	75 (85.2%)
Increase in Hb level ≥ 2 g/dL *n* (%)^∗^		2 (2.2%)	0 (0.0%)	0 (0.0%)	9 (9.6%)	26 (27.4%)	48 (53.3%)	53 (60.2%)	63 (73.3%)	61 (69.3%)
Normalization (women Hb ≥ 12 g/dL, men Hb ≥ 13 g/dL) *n* (%)^∗^		0 (0.0%)	1 (1.1%)	1 (1.1%)	1 (1.1%)	8 (8.4%)	15 (16.7%)	27 (30.7%)	51 (59.3%)	56 (63.6%)
Ferritin (ng/mL)										
*N*	95	—	—	93	—	94	87	88	85	88
Median (Q1‐Q3)	7.00 (5.00–12.90)	—	—	23.20 (15.68–42.38)	—	31.90 (20.00–57.16)	34.00 (22.00–59.00)	35.03 (21.00–53.68)	29.00 (17.00–42.30)	29.65 (14.50–45.00)
Mean ± SD	17.85 ± 49.00	—	—	43.54 ± 62.67	—	50.14 ± 51.25	52.10 ± 61.54	50.91 ± 61.84	41.43 ± 50.74	42.76 ± 59.87
T‐SAT (%)										
*N*	93	91	91	93	93	94	89	87	85	85
Mean ± SD	8.17 ± 9.54	22.13 ± 18.59	22.64 ± 21.57	27.88 ± 31.52	23.84 ± 29.30	19.47 ± 20.14	21.67 ± 18.18	24.40 ± 23.82	22.96 ± 19.74	20.12 ± 14.70
MCV (fL)										
*N*	93	90	90	92	93	93	89	87	85	87
Mean ± SD	70.65 ± 12.09	71.57 ± 9.96	73.03 ± 10.13	74.07 ± 10.71	74.39 ± 9.21	76.02 ± 9.77	77.00 ± 10.69	78.42 ± 9.59	80.80 ± 9.36	81.74 ± 9.62
CRP (mg/L)										
*N*	93	92	92	94	94	95	90	88	86	88
Mean ± SD	3.15 ± 5.08	3.54 ± 6.09	3.38 ± 6.68	3.31 ± 7.13	3.93 ± 6.54	3.85 ± 5.71	4.02 ± 8.67	3.92 ± 7.75	3.47 ± 4.90	3.43 ± 4.57

Abbreviations: CRP, C‐reactive protein; D, day; MCV, mean corpuscular volume; *n*, number of participants; *N*, total number of participants with data available for analysis; V, visit; T‐SAT, transferrin saturation.

^∗^in comparison with Hb level at V2/D0.

After 12 weeks of treatment, nearly all participants had shown a response (Hb increase ≥ 0.5 g/dL: 90.9% of participants, Hb increase ≥ 1 g/dL: 85.2%, Hb increase ≥ 2 g/dL: 69.3% and Hb normal levels: 63.6%), with no significant difference in both geographic zones (data not shown).

Using the threshold of 0.5 g/dL of Hb to define response to treatment, the rate of participants considered responders at D10 was 72.3%. The performance of an Hb increase of 0.5 g/dL at D10 to predict an Hb increase of 1 g/dL at D14 was assessed and the results showed a high sensitivity (95.24%), a satisfactory specificity (74.19%), a high positive predictive value (88.24%) and a high negative predictive value (88.46%). This early identification of response to treatment is confirmed by the median time of 8 days (95% CI [6.0–11.0]) to achieve an increase in Hb of at least 0.5 g/dL and of 15 days (95% CI [11.0–15.0]) to achieve an increase of at least 1 g/dL.

**Figure 3:**
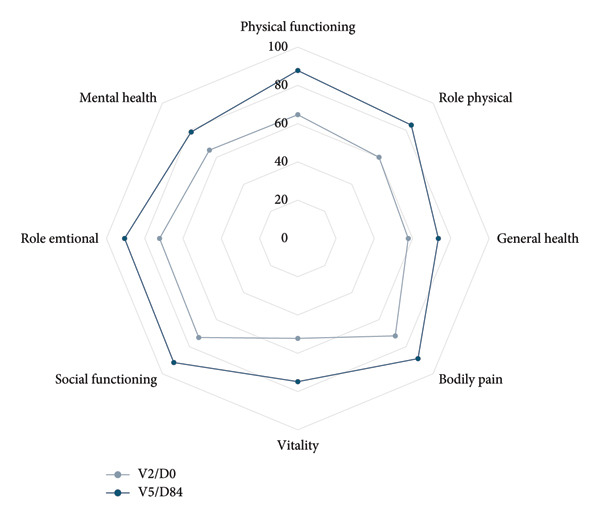
SF‐36 health domains at V2/D0 and V5/D84. The SF‐36 measures eight health domains with 36 questions, transformed to scores ranging from 0, representing poor health status, to 100 representing optimal health status. Changes in health domains are presented for patients included in Europe (SF‐36 questionnaire completed by 51/61 of participants at V2/D0 and 40/59 of participants at V5/D84).

At the end of the study, all symptoms reported at inclusion had reduced: mean change in the fatigue VAS was −2.76 and mean change of SF‐36 summary scores for the physical and mental component were 8.20 and 6.72, respectively. A convergent improvement of at least 10 points was observed for all domains (Figure 3).

Treatment compliance was determined by the number of ampoules of ferrous gluconate taken compared with the theoretical number of ampoules that should have been taken, taking into account all dose modifications. Treatment compliance rate was high (mean: 99.29 ± 4.04). Only three participants in Europe had a compliance rate below 100%.

Overall, 97 participants received at least one dose of treatment. During the study, a total of 34 adverse events (AEs) were reported in 23 participants, including six serious AEs for one participant which were resolving at the end of the study and were deemed unrelated to the treatment. Among all reported AEs, seven AEs were associated with gastrointestinal disorders and three AEs occurring in three participants were considered related to the treatment. These three related AEs included one case of nausea and two cases of bad ampoule taste. All three events were considered non‐serious and mild. The two cases of bad ampoule taste led to a dose reduction as recommended in the protocol. Overall, the study treatment was very well tolerated.

## 4. Discussion

To our knowledge, this study is the first to assess the evolution of biological iron parameters in moderately anaemic patients during the first few days of ferrous gluconate supplementation, with repeated measurements. The FAST study showed that with oral ferrous gluconate treatment, serum iron levels increased rapidly, peaking at D7, and that an increase of at least 0.5 g/dL Hb could be achieved within 9‐10 days of treatment. Rapid normalization of ferritin levels was also observed. It seems that the responder status to ferrous gluconate treatment could be detected on Day 10 of the supplementation.

Data comparing the onset of action among different ferrous salts remain limited. While our study was not designed for direct comparison between iron salts, the observed haematologic response aligns with previous literature suggesting that ferrous gluconate offers similar efficacy to ferrous sulphate. For instance, a randomised clinical trial in children by Falahati et al. demonstrated similar prophylactic effectiveness between ferrous sulphate and ferrous gluconate [17]. Our results complement this by providing real‐world data on the therapeutic use of ferrous gluconate in an adult population with IDA.

In addition, previous studies on the kinetics of efficacy biomarkers of oral iron supplementation have proposed to use an increase of Hb ≥ 1 g/dL at D14 as a marker of response to treatment [16]. In the FAST study, results showed that an Hb increase ≥ 0.5 g/dL as early as D10 could accurately predict a response at D14. These findings suggest that an assessment of Hb evolution as early as the 10^th^ day of treatment could enable to early detect the response to oral iron supplementation. The specificity (74.19%) is comparable to the one reported by Okam et al. (79.3%) for a D14 response threshold [16]. This was considered manageable as continuing oral iron and performing follow‐up procedures in nonresponders would be low risk. Our approach prioritizes high sensitivity (95.24%) to ensure that true responders are not missed.

From a tolerability perspective, ferrous gluconate may offer some advantages over ferrous sulphate. According to guidelines from the British Society of Gastroenterology, alternative iron formulations such as ferrous gluconate or ferrous fumarate may be better tolerated in patients who experience gastrointestinal side effects with ferrous sulphate [23]. In our study, ferrous gluconate was very well tolerated throughout the 3‐month treatment period, with only few adverse effects. This observation reinforces the notion that ferrous gluconate may represent a suitable alternative for patients who are sensitive to the gastrointestinal side effects commonly associated with other oral iron salts. Despite the moderate level of IDA at baseline, serum iron increased as soon as D3 of treatment, with a peak at D7 suggesting a good absorption of oral iron. This could be attributable to the liquid formulation of the ferrous gluconate used in the study, reinforced by copper gluconate, well known for its role in facilitating iron intestinal absorption and metabolism, in addition to the Hb synthesis [24]. Using a Caco‐2 cells system, an *ex vivo* study showed the role of copper in the permeability and the transport of iron gluconate across intestinal epithelium [25]. The rapid increase in reticulocyte count, with a peak at D10, indirectly confirms this good and rapid iron absorption.

The improvement in biological iron parameters under oral iron supplementation is accompanied in this study by a reduction in fatigue and a marked improvement in physical and mental subscales of quality of life. Although these results are consistent with scientific literature showing that iron deficiency is associated with fatigue and impaired quality of life [26], they should be analysed cautiously as this study was open label.

This study also had other limitations. To be included in the study, participants had to be moderately anaemic. Thus, a substantial number of participants had to be screened as the limits of acceptable Hb levels were narrow. The screening process reached its objective of only including patients with moderate IDA, as mean Hb level was 9.13 g/dL at inclusion. The women/men ratio of anaemic patients included in this study was higher than expected according to the global worldwide prevalence of IDA but consistent with the prevalence of IDA reported in the countries where the study was conducted, particularly in Africa [27].

## 5. Conclusion

The FAST study demonstrates for the first time the rapid onset‐of‐action of oral liquid solution of ferrous gluconate in patients with iron deficiency anaemia. This study also suggests that an Hb increase of 0.5 g/dL, detected at Day 10, may serve as an early predictor of subsequent overall Hb response. While this finding is promising, it should be interpreted with caution and not yet considered a definitive clinical threshold. Nevertheless, it may already serve as a valuable tool to help guide clinical decisions and could potentially inform the choice of the route of administration for iron supplementation in IDA patients, particularly when rapid correction of iron deficit is required.

## Ethics Statement

The protocol was approved by the competent authorities of France (Agence Nationale de Sécurité du Médicament et des produits de santé), Bulgaria (Bulgarian Drug Agency) and Kenya (Pharmacy and Poisons Board) and by independent ethics committee from the three countries (Comité de Protection des Personnes, Poitiers Ouest III, France; Ethics Committee for Clinical Trials, Bulgaria; Jaramogi Oginga Odinga Teaching and Referral Hospital, Institutional Scientific and Ethical Research Committee, Kenya). The study was conducted in accordance with the protocol, the Declaration of Helsinki and its amendments, the applicable International Conference of Harmonization E6 (R2) Good Clinical Practices Guidelines and the applicable local regulations.

## Consent

All participants provided written informed consent before study inclusion.

## Disclosure

The study was designed and conducted according to the protocol by the sponsor, Laboratoire Innotech International. All authors approved the manuscript for submission.

## Conflicts of Interest

Julie Escola, Océane Gobert, Anne‐Laure Kerveillant and François Verrière are employees of Innotech International; Julie Trichereau is an employee of the CRO contracted by Innotech International; Patrice Cacoub reported links of interest with Innotech International, Vifor and Servier; Fredrick O. Otieno reported links of interest with Innotech International.

## Author Contributions

All authors contributed to data interpretation and manuscript development and vouch for the accuracy and completeness of the reported data.

## Funding

The study was sponsored​ by Laboratoire INNOTECH INTERNATIONAL.

## Data Availability

Anonymized data not published within this article will be made available on request from the corresponding author.
